# Vitamin D status in hospitalized COVID‑19 patients is associated with disease severity and IL-5 production

**DOI:** 10.1186/s12985-023-02165-1

**Published:** 2023-09-13

**Authors:** Yali Qiu, Wuping Bao, Xue Tian, Yingying Zhang, Yilin Pan, Guogang Xie, Aihua Bao, Dongning Yin, Min Zhang, Yan Zhou

**Affiliations:** 1 Department of Respiratory and Critical Care Medicine, Shanghai General Hospital of Nanjing Medical University, Shanghai, China; 2 Department of Respiratory and Critical Care Medicine, Changzhou Medical Center, Changzhou Third People’s Hospital, Nanjing Medical University, Changzhou, China; 3 Department of Respiratory and Critical Care Medicine, Shanghai General Hospital, Shanghai Jiao Tong University School of Medicine, Shanghai, China

**Keywords:** Vitamin D, 25-hydroxyvitamin D, IL-5, SARS-CoV-2 infection, COVID-19

## Abstract

**Background:**

There are many studies on the relationship between vitamin D and coronavirus disease 2019 (COVID-19), while the results are matters of debate and the mechanisms remain unknown. The present study was performed to assess the impact of serum 25-hydroxyvitamin D [25(OH)D] levels on the severity of disease in hospitalized COVID-19 patients and identify potential mechanisms of 25(OH)D alterations.

**Methods:**

A total of 399 hospitalized COVID-19 patients were recruited from three centers between December 19, 2022, and February 1, 2023. Medical history, laboratory examination, and radiologic data were retrospectively collected. The patients were divided into four groups based on disease severity. Serum 25(OH)D levels in the patients were determined by the electrochemiluminescence method and cytokines were detected by flow cytometry. The relationship between serum 25(OH)D status and the severity of COVID-19, and the correlation between 25(OH)D levels and cytokines in COVID-19 patients were assessed.

**Results:**

Levels of 25(OH)D were significantly lower in the deceased group than in the other three groups (*P* < 0.05), and lower in the critical group than in the general group (*P* < 0.05). There were no significant differences in the 25(OH)D levels between the general and severe groups (*P* > 0.05). The levels of 25(OH)D (odds ratio = 0.986, 95% confidence interval: 0.973–0.998, *P* = 0.024) and IL-5 (odds ratio = 1.239, 95% confidence interval: 1.104–1.391, *P* = 0.04) were independent risk factors for the severity of COVID-19 disease upon admission. Serum 25(OH)D levels were able to predict the mortality of patients with COVID-19, and the predictive value was even higher when combined with IL-5 levels and eosinophil (Eos) count. Circulating 25(OH)D status correlated negatively with the expression of IL-5 (r=-0.262, *P* < 0.001) and was positively linked with CD8^+^ T cell counts (r=-0.121, *P* < 0.05) in patients with COVID-19.

**Conclusions:**

This study found that the serum 25(OH)D status combined with IL-5 levels and Eos counts could be identified as a predictive factor for recognizing the risk of COVID-19 mortality. The serum 25(OH)D status in COVID-19 patients correlated negatively with the expression of IL-5. The potential mechanism for this relationship is worth further exploration.

## Introduction

The COVID-19 outbreak began in March 2020 and spread globally, affecting millions of people. SARS-CoV-2, the causative agent of COVID-19, binds to angiotensin converting enzyme 2 (ACE2) and enters the host cell, leading to the development of pulmonary lesions and pneumonia [1, 2]. Research into effective treatments need to be coupled with vaccine development [3, 4]. Drugs that specifically target SARS-CoV-2 remain lacking. Antiviral medications such as paxlovid and VV116 are commonly used in clinics, however, the efficacy and safety of these drugs require further clinical validation. Evolving COVID-19 variants are gaining higher infectivity and greater capacity to evade antibody protection, limiting the ability of the current vaccine to prevent infection. While COVID-19 control still faces multiple challenges in the short term, there is an urgent need to develop targeted drugs against SARS-CoV-2 [5].

COVID-19 mortality varies by geographic region. One mostly overlooked factor that may impact regional differences is the relative vitamin D status of populations with differing amounts of available sunlight [6]. Since the mid-1980s, more attention has been focused on vitamin D due to its ability to prevent disease by modulating innate and adaptive immune responses [7, 8]. Recently, vitamin D was shown to also impact the renin-angiotensin system (RAS), particularlyACE2, the primary host cell receptor for SARS-CoV-2 [9]. Vitamin D also plays an important role in regulating viral infections by inducing cathelicidins and defensins, which reduce viral replication [10]. Vitamin D includes two liposoluble compounds, vitamin D_2_ (ergocalciferol) and vitamin D_3_ (cholecalciferol), and is primarily formed by the absorption of sunlight by the skin and a small percentage from diet [11]. The European Calcifediol Tissue Society Working Group defined severe vitamin D deficiency as a serum 25(OH)D level < 30 nmol/L [12]. It is estimated that more than one billion people worldwide have vitamin D deficiency [13].

During COVID-19, vitamin D deficiency may alter virus-specific immune responses, including T cell function [14], and promote adverse health outcomes in critically ill patients [15]. The functional depletion of CD8^+^ T cells are associated with severe SARS-CoV-2 infection [16]. Interestingly, COVID-19 patients who develop severe disease have a complex maladapted immune profile that is accompanied by an increase in cytokines, such as IL-6, along with higher type-1 (e.g. IL-12) and type-2 cytokine (e.g. IL-5) levels [17]. Wang et al. [18] found that vitamin D supplementation can reduce IL-5 levels in patients with asthma and chronic obstructive pulmonary disease (COPD). Whether vitamin D plays a protective role by regulating IL-5 in COVID-19 patients remains unknown.

To date, studies on the relationship between vitamin D and COVID-19 are controversial, and the mechanisms remain unknown. Herein, we conducted a retrospective, multi-center, cross-sectional, observational study to analyze vitamin D levels in hospitalized COVID-19 patients with different clinical classifications. Our study further assessed whether particular characteristics were associated with vitamin D deficiency. Our study focus on finding [19] the association of vitamin D levels with the severity of COVID-19 in patients and whether the serum 25(OH)D status combined with IL-5 levels and Eos counts could be predict the risk of COVID-19 mortality. Serum 25(OH)D levels were negatively correlated with IL-5 in COVID-19 patients, which laid a foundation for further research on the mechanisms involved in these findings.

## Materials and methods

### Participants

**Fig. 1:**
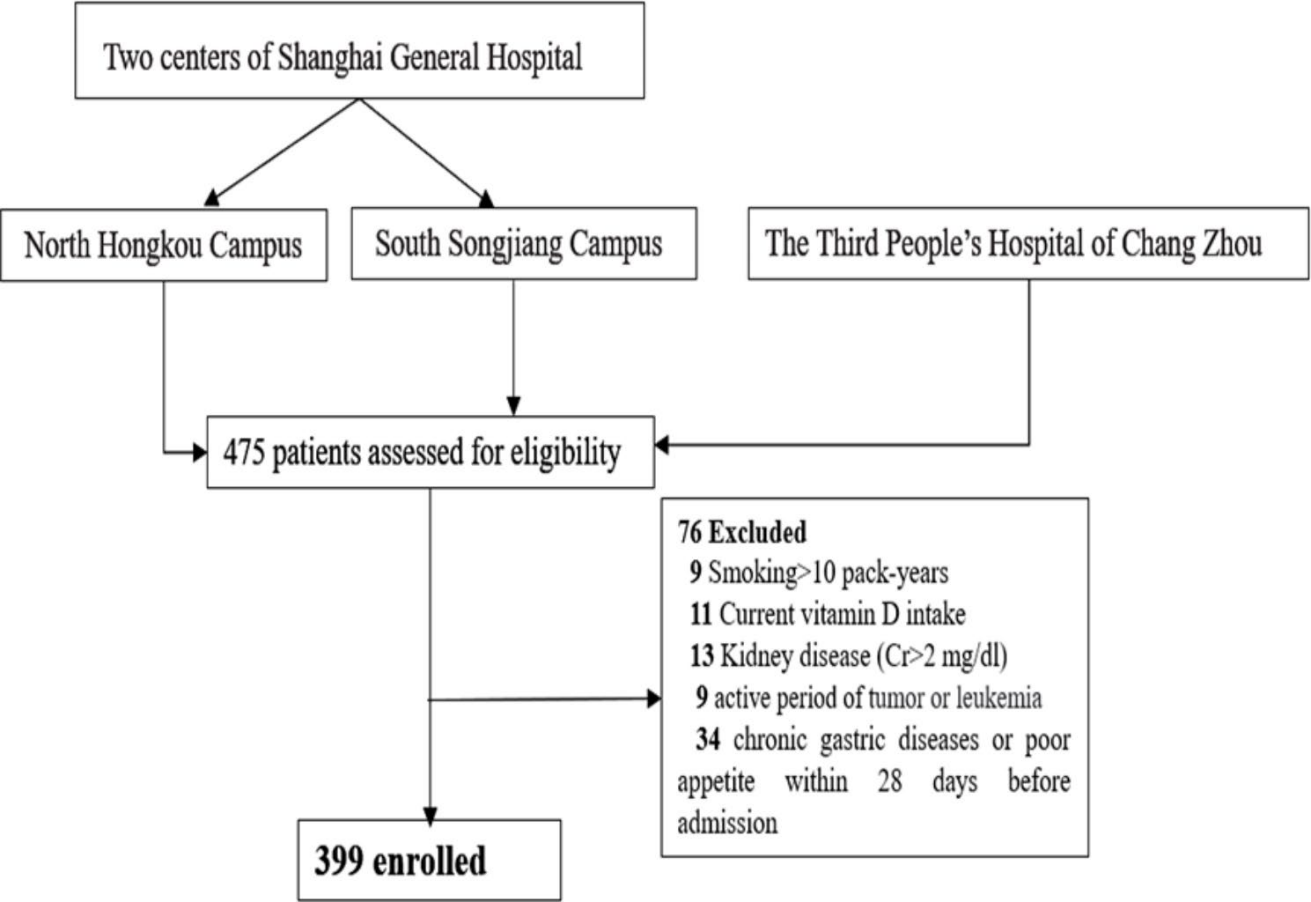
Flow diagram for patients with coronavirus disease 2019 (COVID-19) included in the study

A total of 475 participants were enrolled from hospitalized patients in three centers: One center is in an urban area on the North Hongkou Campus and the second center is in a suburban area on the South Songjiang Campus. For the validation analysis, patients were recruited from an additional hospital, the Third People’s Hospital of Chang Zhou, in the Jiangsu region. The final analysis included data from 399 hospitalized COVID-19 patients recruited between December 30, 2022 and February 1,, 2023 (Fig. 1). All hospitalized patients received a standard diagnosis protocol based on “The Tenth Edition of the Prevention and Control Guidance for COVID-19” published by the National Health Commission of China. Patients of both sexes who were ≥ 18 years of age were included in this study. Those who had undergone bariatric surgery, chronic gastric diseases or poor appetite within 28 days before admission, neoplasms, abnormal kidney function, debilitating immune-related diseases, or a final clinical diagnosis that was not COVID-19, were excluded from the study. Patients with mild COVID-19 infection who did not require hospitalization were also excluded.

This was a retrospective cohort study that was approved by the Ethics Committees at Shanghai General Hospital and the Third People’s Hospital of Chang Zhou (02 A-A20210008). The study was conducted in accordance with the relevant guidelines and regulations/ethical principles of the Declaration of Helsinki. A waiver of informed consent was obtained from the study participants. Data collection began as soon as ethical approval was obtained and was completed once the investigators felt that the first peak of COVID-19 admissions had passed.

### Study protocol

Laboratory testing for COVID-19 was conducted by throat swab, and samples were tested for SARS-CoV-2 RNA using real-time PCR. A clinical diagnosis of COVID-19 was made if laboratory testing was negative but patients had signs and symptoms of SARS-CoV-2 infection, including a persistent cough, dyspnea, fever, low oxygen saturation (SpO_2_), and bilateral interstitial infiltrates on a computed tomography (CT) scan or chest radiograph. Biochemical examinations and CT scans were given routinely upon admission. Demographic, clinical, and epidemiological data were retrospectively collected from electronic medical records and laboratory information management systems. All data were recorded and checked separately by two qualified researchers.

Patients infected with COVID-19 were subdivided into a (1) general group [infected with COVID-19, persistent fever and/or cough, dyspnea, a respiratory rate (RR) < 30 beats per minute and SpO_2_ > 93%], (2) severe group [infected with COVID-19, RR ≥ 30 beats per minute or SpO_2_ ≤ 93% or arterial oxygen pressure (PaO_2_)/ concentrate of oxygen inhalation (FiO_2_) ≤ 300], (3) critical group [infected with COVID-19, respiratory failure and requiring mechanical ventilation or shock or other organ failure requiring intensive care unit (ICU) monitoring] and (4) deceased group (died as a result of COVID-19 infection). The critical group included those who later died from the COVID-19 infection.

According to Russian and international guidelines [20], normal vitamin D status is defined as 25(OH)D ≥ 30 ng/mL (≥ 75 nmol/L), insufficient vitamin D status is defined as 25(OH)D 20–29 ng/mL (50–74 nmol/L), and deficient vitamin D status is defined as < 20 ng/mL (< 50 nmol/L).

### Determination of serum 25-hydroxyvitamin D concentration

The levels of serum 25(OH)D were determined by using the electrochemiluminescence method in an immunoenzymatic assay, using standardized reagents on a COBAS 8000 Modular Analyzer (Roche, Basel, Switzerland) at two centers of Shanghai General Hospital and the Third People’s Hospital of Chang Zhou [21].

### Cytokines were detected by flow cytometry

Peripheral blood (PB) samples were diluted in RPMI-1640 medium to a final concentration of 2 × 10^6^ cells/mL. PB cells were then stained with a fixable viability dye (eBioscience) and antibodies for surface labeling for 30 min at 4 °C, before fixation with 4% formaldehyde for 10 min at room temperature in the dark. After fixation, cells were incubated with permeabilization buffer (eBioscience) for 10 min at room temperature in the dark and stained for cytokines for 30 min at 4 °C. After intracellular staining, cells were washed with FACSFlow and were resuspended in FACSFlow for flow cytometric analysis [22].

### Statistical analysis

All analyses were performed using SPSS 25.0 software (Armonk, NY, USA) and GraphPad Prism 8.0 (La Jolla, CA, USA). Continuous variables were expressed as the mean ± standard deviation (SD) or median and interquartile range. One-way analysis of variance (ANOVA) and the Student–Newman–Keuls (S-N-K) post hoc test were used to compare multiple groups. Differences between two groups were compared with the Kruskal-wallis test based on the distribution of the data. Categorical variables were presented as numbers and percentages and compared using the chi-squared or Fisher’s exact tests as appropriate. Correlation analysis was performed using Spearman’s correlation test. Multivariate ordered logistic regression analysis (adjusting for age, IL-5, CD8 + T cells and comorbidities), was conducted to identify independent risk factors for severity of COVID-19. Receiver operating characteristic (ROC) curve analysis was performed to evaluate COVID-19-specific mortality. A two-sided *P*-value < 0.05 was considered statistically significant.

## Results

### Baseline characteristics of COVID-19 patients

A total of 399 adult COVID-19 patients were included in the study. The main demographic, epidemiological, clinical characteristics, and disease-severity data of the patients in the four study groups are summarized in Table 1. The final analysis included 247 men (62%) and 152 women (38%), aged 72.98 ± 0.64 years.

**Table 1:** Differences in characteristics of patients based on the different severity of COVID-19 at admission

Variables	General (n = 175)	Severe (n = 149)	Critical (n = 75)	Deceased (n = 42)		*P* value
Age (years)	70.52 ± 1.04	73.49 ± 0.97	77.52 ± 1.14		79.88 ± 1.26	**< 0.01**
BMI	23.95 ± 0.34	23.94 ± 0.30	23.59 ± 0.38		23.42 ± 0.49	0.79
Sex (male), n (%)	108 (62)	86 (59)	53 (70)		26 (62)	0.36
Current smoker, n (%)	28 (16)	27 (18)	14 (19)		7(17)	0.94
**Comorbidity**						
Diabetes, n (%)	41(23)	47(32)	31(41)		15(36)	**0.03**
Hypertension, n (%)	88 (50)	95(64)	52(69)		33(79)	0.001
Coronary Heart Disease n (%)	40(23)	45(30)	25(33)		16(38)	0.06
Chronic lung disease (%)	24 (14)	20 (13)	11 (15)		7 (17)	0.89
CKD (%)	13 (7)	15(10)	12 (16)		9 (21)	**0.04**
**Symptoms**						
Duration of Fever, D	8.24 ± 0.55	9.02 ± 0.60	10.83 ± 0.85		11.03 ± 0.69	**0.03**
Dyspnea, %	108 (62)	102 (68)	75 (100)		42 (100)	**0.00**
**Laboratory findings**						
Serum 25(OH)D level (ng/mL)	43.16 ± 1.82	39.44 ± 1.88	35.64 ± 2.32		28.02 ± 1.99	**< 0.001**
Neutrophils, E + 09/L	5.34 ± 0.25	5.93 ± 2.88	7.62 ± 0.57		8.83 ± 0.69	**< 0.001**
Lymphocytes, E + 09/L	1.19 ± 0.54	1.10 ± 0.15	0.64 ± 0.39		0.61 ± 0.49	**< 0.01**
Eosinophil, E + 09/L	0.06 ± 0.01	0.03 ± 0.0	0.01 ± 0.00		0.01 ± 0.00	**< 0.01**
CRP, mg/L	34.09 ± 3.58	55.54 ± 5.38	90.0 ± 7.64		97.34 ± 11.35	**< 0.001**
PCT, ng/mL	1.46 ± 0.78	1.33 ± 0.73	0.81 ± 0.31		0.80 ± 0.39	0.93
D-dimer, µg/mL	1.57 ± 0.30	2.45 ± 0.38	4.15 ± 0.84		5.94 ± 1.59	**< 0.001**
Uric acid, µmol/L	271.71 ± 14.11	289.57 ± 18.82	292.10 ± 32.74		297.87 ± 43.58	0.53
Ferritin, ng/mL	447.66 ± 27.39	504.72 ± 29.80	575.0 ± 49.45		626.86 ± 71.96	**0.02**
LDH, U/L	236.39 ± 14.31	276.35 ± 20.67	375.65 ± 40.59		479.8 ± 82.99	**< 0.001**
Glucose, mmol/L	6.79 ± 0.27	7.80 ± 0.29	8.39 ± 0.42		8.17 ± 0.67	**< 0.001**
IL-5, pg/mL	1.65 ± 0.31	2.29 ± 0.15	3.36 ± 0.44		3.22 ± 0.75	**0.003**
IL-6, pg/mL	12.20 ± 1.68	20.57 ± 3.20	30.67 ± 6.55		35.22 ± 11.36	**0.002**
IL-10, pg/mL	3.15 ± 0.53	3.38 ± 0.34	3.58 ± 0.28		3.47 ± 0.47	0.93
CD4^+^T cells	344.16 ± 23.93	256.85 ± 19.61	170.16 ± 2187		121.37 ± 15.11	**< 0.001**
CD8^+^T cells	271.31 ± 18.89	197.56 ± 23.90	123.45 ± 13.63		123.31 ± 19.47	**< 0.001**
CD4^+^/CD8^+^	1.58 ± 0.12	1.82 ± 0.11	2.32 ± 0.28		1.89 ± 0.32	**0.02**
B cell counts	132.52 ± 13.02	108.98 ± 9.52	82.78 ± 11.56		56.37 ± 11.24	**0.005**

Abbreviations: BMI, body mass index; CKD, Chronic Kidney Disease; CRP, C-reactive protein; LDH, Lactate dehydrogenase; PCT, procalcitonin; 25(OH)D, the major circulating form of vitamin D. *P* < 0.05 values are bolded

There were significant differences in neutrophil, lymphocyte, eosinophil (Eos), C-reactive protein (CRP), D-dimer, lactate dehydrogenase (LDH), glucose, IL-6, ferritin, CD4 + T cell, CD4+/CD8 + T cell, CD8 + T cell, and B cell counts in the serum of the four COVID-19 patient groups (*P* < 0.05). Procalcitonin (PCT), uric acid, and IL-10 levels remained similar in the serum of the patients in each group (*P* > 0.05). Patients in the critical group had elevated baseline or maximum serum CRP, IL-6, LDH, ferritin, and D-dimer levels and lower CD4 + T cell, CD8 + T cell, and B cell counts, which was characterized as an obvious inflammatory response. Serum 25(OH)D and IL-5 levels also differed significantly between the four groups (*P* < 0.01). IL-5 expression was significantly elevated in the critical and deceased groups (*P* < 0.01).

**Fig. 2:**
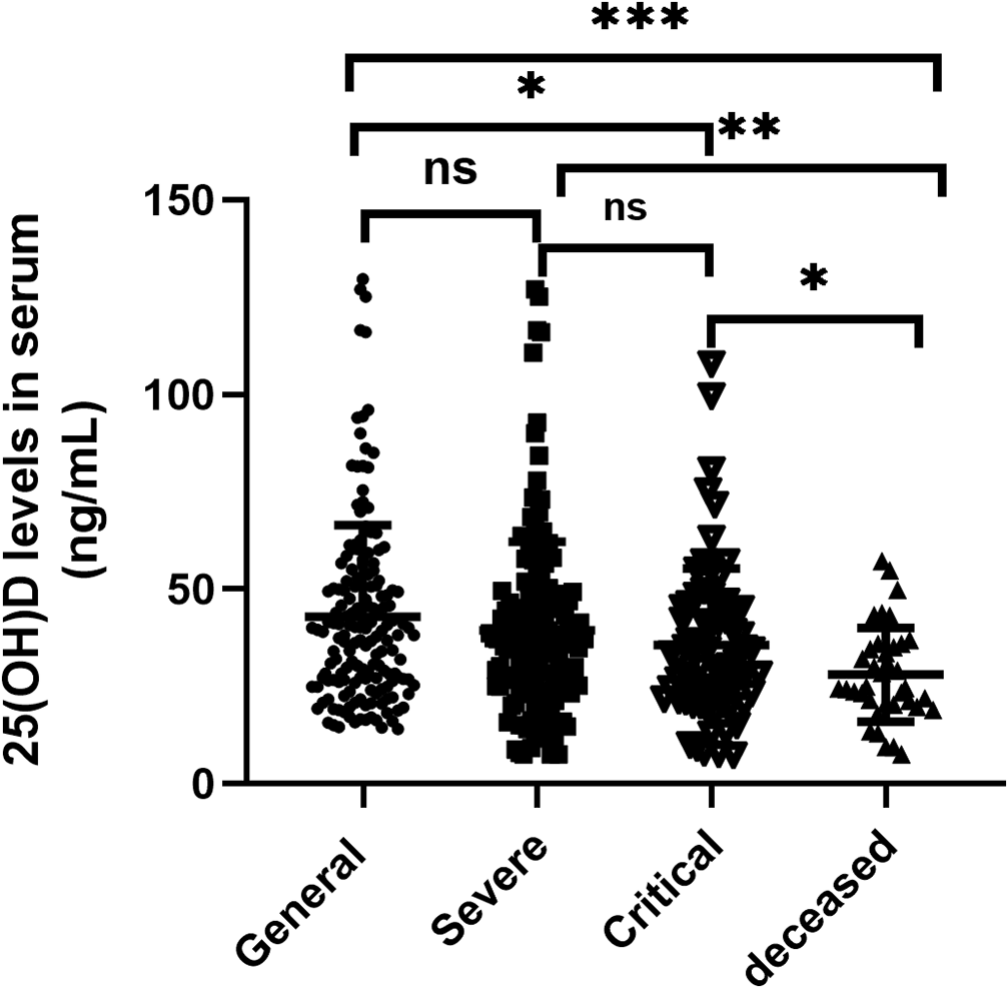
Serum 25(OH)D status in patients with different degrees of COVID-19. The 25(OH)D status was significantly reduced in the deceased group compared with that in the other three groups, and the mean levels in the critical group were lower than the general group. The levels of 25(OH)D in serum between the general group and the severe group were no significant differences to be found. *P < 0.05, **P < 0.01, **P < 0.001

Serum 25(OH)D levels were significantly lower in the deceased group than in the other three groups (*P* < 0.05), and the mean levels were lower in the critical group than in the general group (*P* < 0.001). There were no differences in the levels of 25(OH)D between the general and severe groups, and the severe group also showed no significant differences from the critical group (*P* > 0.05, Fig. 2).

### Clinical characteristics of COVID-19 patients by 25(OH)D status

COVID-19 patients were divided into three groups based on circulating serum 25(OH)D levels at hospital admission (Table 2). A total of 29 patients (7.3%) had normal 25(OH)D status, while 15.3% and 77.4% had insufficient and deficient status, respectively. Serum 25(OH)D levels were lower in patients in the critical group than those in the general group. These findings indicated that vitamin D deficiency was associated with disease severity. Indeed, 20% and 18% of vitamin D deficient and insufficient patients, respectively, had critical disease. While the body mass index (BMI) of patients in the normal 25(OH)D group was higher than those in the vitamin D insufficient and deficient groups, this result was not statistically significant (*P* > 0.05). The proportion of COVID-19 patients with diabetes was significantly higher in the vitamin D deficient group (*P* < 0.05). However, there were no significant differences in the prevalence of coronary heart disease and hypertension among patients with different levels of 25(OH)D (*P* > 0.05).

**Table 2:** Differences in characteristics of COVID-19 patients based on different vitamin D status groups at admission

Variables	Normal (n = 29)	Insufficiency (n = 61)	Deficiency (n = 309)	*P* value	
Age (years)	72.80 ± 1.13	71.46 ± 1.07	74.29 ± 1.04		0.14
BMI	25.76 ± 0.37	23.92 ± 0.40	23.73 ± 0.24		0.06
Sex (male), n (%)	15 (52)	44 (72)	188 (61)		0.13
Current smoker, n (%)	6 (21)	13 (21)	50 (17)		0.55
**Comorbidity**					
Diabetes, n (%)	6(21)	16(26)	97(31)		**0.00**
Hypertension, n (%)	16(55)	35(57)	184 (60)		0.12
Coronary Heart Disease n (%)	8(28)	19(31)	83(27)		0.09
Chronic lung disease (%)	3 (10)	9 (15)	43 (14)		0.83
CKD (%)	2 (7)	3(5)	35(11)		0.22
**Laboratory findings**					
Neutrophils, E + 09/L	5.51 ± 0.65	5.21 ± 0.45	6.21 ± 0.22		0.141
Lymphocytes, E + 09/L	1.19 ± 0.54	1.10 ± 0.15	0.64 ± 0.39		0.656
Eosinophil, E + 09/L	0.05 ± 0.05	0.05 ± 0.01	0.04 ± 0.00		0.837
CRP, mg/L	45.61 ± 10.25	43.78 ± 7.04	53.59 ± 3.55		0.087
PCT, ng/mL	0.33 ± 0.16	0.24 ± 0.07	0.92 ± 0.31		0.546
D-dimer, µg/mL	0.97 ± 0.16	1.34 ± 0.26	2.27 ± 0.21		**0.048**
Uric acid, µmol/L	265.93 ± 18.21	263.74 ± 10.65	291.95 ± 8.19		0.246
Ferritin, ng/mL	464.73 ± 20.72	549.64 ± 51.29	612.05 ± 89.18		0.06
LDH, U/L	264.43 ± 13.40	252.34 ± 17.52	281.44 ± 7.76		0.270
Glucose, mmol/L	6.62 ± 0.25	7.53 ± 0.22	8.11 ± 0.57		0.119
IL-5, pg/mL	2.45 ± 0.20	2.59 ± 0.07	3.92 ± 0.29		**0.045**
IL-6, pg/mL	10.97 ± 2.27	14.86 ± 2.73	21.75 ± 2.98		0.373
IL-10, pg/mL	4.90 ± 2.17	3.20 ± 0.34	3.25 ± 0.28		0.318
CD4^+^T cells	292.75 ± 16.53	240.11 ± 25.94	223.70 ± 37.57		0.217
CD8^+^T cells	218.90 ± 13.53	167.55 ± 17.85	137.55 ± 30.02		**0.034**
CD4^+^/CD8^+^	2.08 ± 0.28	1.64 ± 0.17	1.84 ± 0.11		0.56
B cells count	110.31 ± 21.16	112.09 ± 12.63	112.66 ± 8.26		0.809

BMI, body mass index; CKD, chronic kidney disease; CRP, C-reactive protein; LDH, Lactate dehydrogenase; PCT, procalcitonin; 25(OH)D, the major circulating form of vitamin D. *P* < 0.05 values are bolded

D-dimer, IL-5, and CD8^+^ T cell levels differed significantly between the three 25 (OH)D groups (*P* < 0.05). D-dimer and IL-5 levels were higher in patients with vitamin D deficiency than in those in the normal and vitamin D insufficient groups (*P* < 0.05), while CD8 + T cell numbers were significantly lower in the vitamin D deficient group (*P* > 0.05).

### Serum 25(OH)D and IL-5 levels are risk factors for COVID-19 disease severity upon admission

Multivariate ordinal logistic regression analysis found that age, LDH, and CD8^+^ T cell counts were associated with COVID-19 disease severity (Table 3). Serum 25(OH)D (OR = 0.986, 95%CI = 0.973–0.998, *P* = 0.024) and IL-5 (OR = 1.239, 95%CI = 1.104–1.391, *P* = 0.000) levels were also independent risk factors for disease severity upon admission. To adjust for confounding factors, we adjusted for age, sex, IL-5, CD8 + T cells and comorbidities. Serum 25(OH)D levels (OR = 0.987, 95% CI = 0.998–0.977, *P* = 0.022) and IL-5 (OR = 1.207, 95%CI = 1.327–1.098, *P* = 0.000) were strongly associated with COVID-19 severity (Table 3).

**Table 3:** Risk factors for severity of Patients with COVID-19 at admission

Variables	Unadjusted model		Adjusted model	
	OR (95%CI)	*P-*value	OR (95%CI)	*P-*value
Age (years)	1.024 (1.001–1.048)	**0.044**	1.035 (1.056–1.014)	**0.001**
Male	1.714 (0.967–3.037)	0.065	1.970 (3.178–1.222)	**0.005**
**Comorbidity**				
Diabetes, n (%)	0.759 (0.379–1.519)	0.436	0.708 (1.203–0.417)	0.202
Hypertension, n (%)	0.598 (0.339–1.055)	0.076	0.541 (0.884 − 0.331)	**0.014**
Coronary Heart Disease n (%)	0.730 (0.384–1.387)	0.336	0.945 (1.629–0.549)	0.839
**Laboratory findings**				
Serum 25(OH)D level (ng/mL)	0.986 (0.973–0.998)	**0.024**	0.987 (0.998 − 0.977)	**0.022**
Neutrophils, E + 09/L	1.024(0.941–1.114)	0.582		
Lymphocytes, E + 09/L	0.984 (0.837–1.154)	0.830		
Eosinophil, E + 09/L	0.005 (2.024E-5-1.081)	0.053		
CRP, mg/L	1.004 (1.000-1.009)	0.057		
D-dimer, µg/mL	1.024 (0.976–1.076)	0.333		
Ferritin, ng/mL	1 (0.999–1.001)	0.53		
Uric acid, µmol/L	1.001 (0.998–1.003)	0.597		
LDH, U/L	1.005 (1.002–1.007)	**0.000**		
Glucose, mmol/L	0.975 (0.893–1.065)	0.574		
IL-5, pg/mL	1.239 (1.104–1.391)	**0.000**	1.207 (1.327–1.098)	**0.000**
IL-6, pg/mL	0.999 (0.988–1.010)	0.999		
IL-10, pg/mL	1.011 (0.945–1.083)	0.743		
CD4^+^T	1 (0.998–1.002)	0.881		
CD8^+^T	0.998 (0.996-1)	**0.039**	0.997 (0.998 − 0.995)	**0.000**
B cells count	1 (0.997–1.003)	0.852		

Abbreviations: LDH, Lactate dehydrogenase. CI, 95% confidence interval; CRP, C-reactive protein.

Model is adjusted for age, sex, IL-5, CD8 + T cells and comorbidities. Data are presented as frequencies (%) and odds ratio (95% CI). OR, odds ratio; CI, confidence interval. *P* < 0.05 values are bolded.

### Serum 25(OH)D, IL-5, and Eos levels were strong predictors of COVID-19 patient mortality

**Fig. 3:**
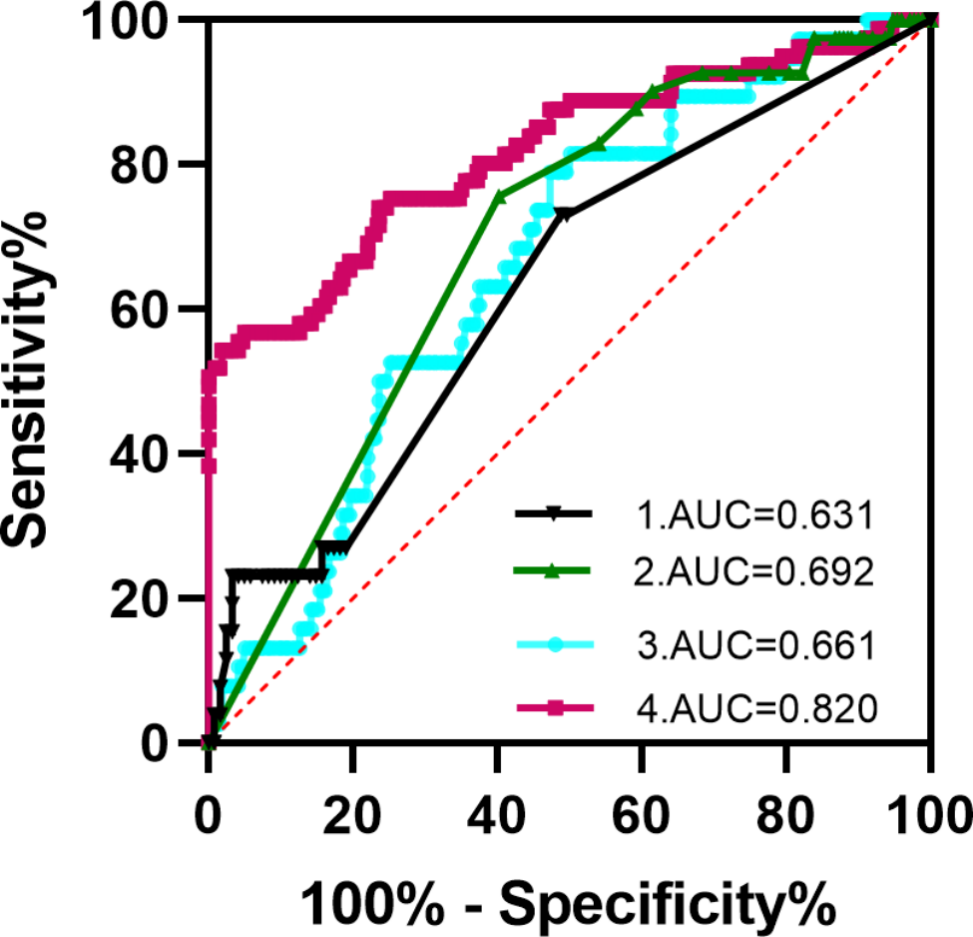
ROC curve analysis was performed to evaluate the mortality of COVID-19. The expression of IL-5 (1) had AUC of 0.6310 and the cutoff value was 1.70; the Eos counts (2) had AUC of 0.692, and the cutoff value was 0.015; while 25(OH)D level (3) had AUC of 0.661 and the cutoff value was 36.04 for the mortality of COVID-19. The combination of the 25(OH)D status with IL-5, the Eos counts (4) had a better value (AUC = 0.820) for the mortality of COVID-19 than either indicator

Serum 25(OH)D, IL-5, and Eos levels of patients in the general (n = 175) and deceased groups (n = 42) were analyzed and the predictive values were evaluated using ROC curves. IL-5 expression had an Area Under the Curve (AUC) of 0.6310 and a cutoff value of 1.70 for COVID-19 mortality, while 25(OH)D levels had an AUC of 0.661 and a cutoff value of 36.04 for COVID-19 mortality. The Eos counts had AUC of 0.692, and the cutoff value was 0.015 for COVID-19 mortality. Serum 25(OH)D, IL-5 and Eos levels together had a better diagnostic value (AUC = 0.820) for COVID-19 severity than any indicator alone (Fig. 3; Table 4).

**Table 4:** Predictive values of IL-5, Eos, 25(OH)D levels and their combination in motality of COVID-19

Characteristic variables	AUC	Cut off Values ^a^	Sensitivity %	Specificity %	*P* value
IL-5	0.631	1.70	73.08	51.24	0.037
Eos	0.692	0.015	75.61	59.77	0.0001
25(OH)D	0.666	36.04	78.95	52.68	0.0014
25(OH)D+ IL-5 + Eos	0.820	---	54.32	98.11	< 0.0001

^a^ The cutoff points were selected by maximizing the sum of sensitivity and specificity. Eos, Eosinophil cell count, IL-5, interleukin-5

### Serum 25(OH)D levels correlated negatively with IL-5 production in COVID-19 patients

**Fig. 4:**
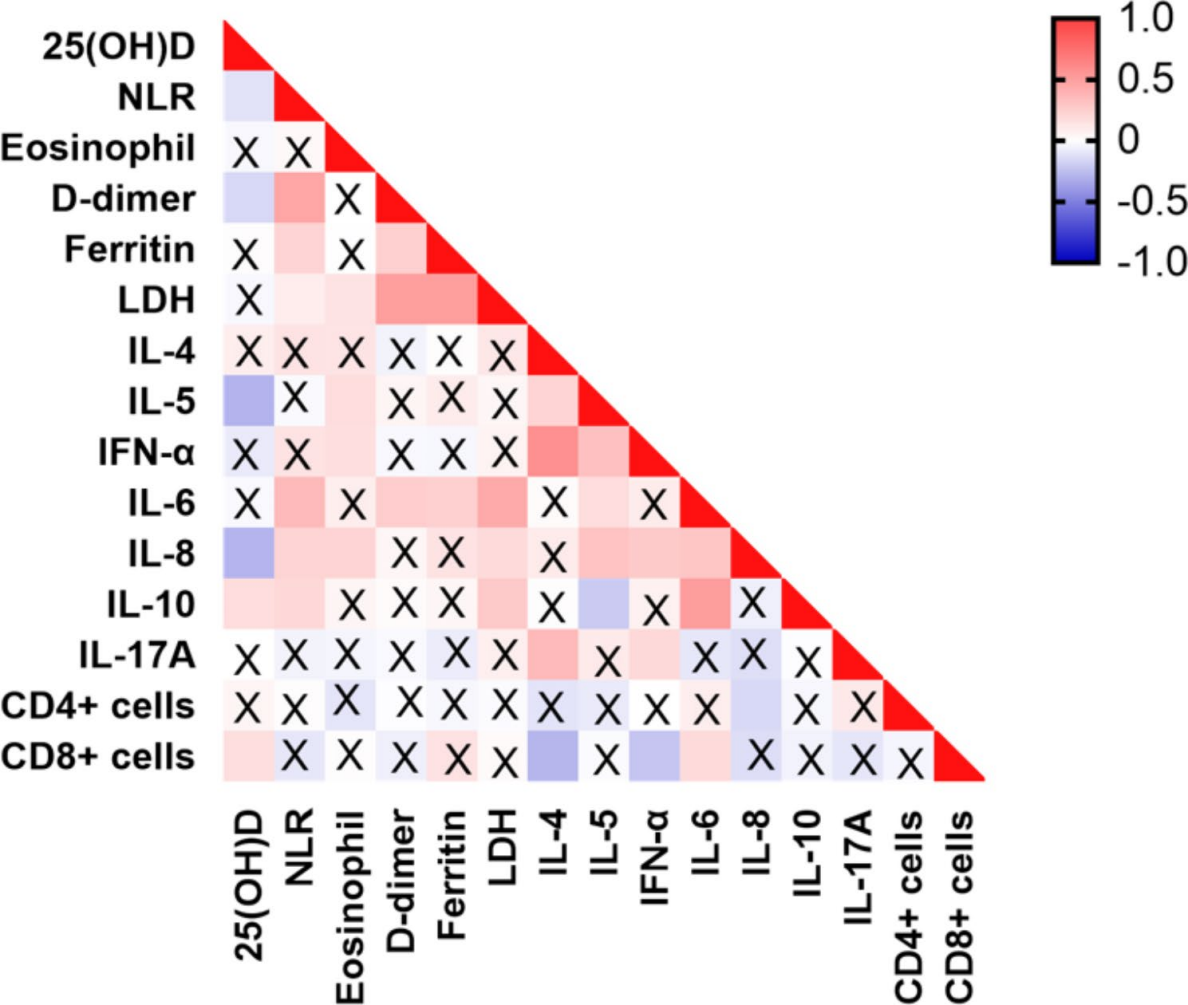
Heatmap of Spearman’s correlation between 25(OH)D status and inflammatory markers in serum of COVID-19 patients. The levels of 25(OH)D in the serum negatively correlated with NLR, D-dimer, IL-5, IL-8, and positively linked with IL-10 and CD8 + T cell counts in patients with COVID-19. The Spearman rank correlation test was conducted. A cross represents no statistical significance. NLR, neutrophil-to-lymphocyte ratio

An association between 25(OH)D values and the inflammatory indicators, LDH, CD4^+^ T cell, and CD8^+^ T cell counts were assessed using Spearman’s rank correlation test (Fig. 4). Serum 25(OH)D levels correlated negatively with the neutrophil-to-lymphocyte ratio (NLR) (r = -0.117, *P* = 0.022), D-dimer (r = − 0.157, *P* = 0.002) and IL-5 level (r = -0.298, *P* < 0.001). While the correlation coefficient was low, vitamin D had a significantly higher correlation with IL-5 than NLR and D-dimer. However, the link between serum 25(OH)D and lymphocyte, eosinophil, LDH, and IL-6 levels was nonsignificant. Serum 25(OH)D levels correlated positively with IL-10 (r = 0.137, *P* = 0.022) and the CD8^+^ T cell count (r = 0.126, *P* = 0.036), but was not significantly linked to the CD4^+^ T cell count.

## Discussion

While several studies [3, 23–25] have assessed the relationship between vitamin D and COVID-19, the results vary. Hernandez, et al. [26] found that 25(OH)D levels were lower in hospitalized COVID-19 patients than in population-based controls, but not find any relationship between vitamin D concentrations and the severity of the disease. However, in a retrospective cohort study [27], COVID-19 mortality was significantly correlated with vitamin D status in different populations. In our study, we found that most COVID-19 patients had vitamin D deficiency, and vitamin D status was associated with the severity of COVID-19 during the acute infection phase. Serum 25(OH)D levels combined with IL-5 levels and Eos counts could serve as predictors of early COVID-19-related mortality. However, the potential mechanism requires further exploration.

COVID-19 disease severity and death correlate with high levels of acute phase reactants [28]. These results agree with the findings of Favaloro et al. [29] that showed that elevated LDH, D-dimer, CRP, and IL-6 levels at the time of diagnosis are linked to severe outcomes. In the current study, levels of the primary lymphocyte subsets were lower in critical and deceased patients with COVID-19, with far below normal T cell and B cell counts. Chen et al. [30] found that the concentrations of PCT and high-sensitivity CRP were significantly higher in deceased patients. The PCT levels were lower in deceased patients in the current study, however, these were not statistically significant. Serum 25(OH)D status was also significantly lower in the deceased group than in the other groups, and the expression of IL-5 was higher in both the critical and deceased groups than in the general and severe groups. Low serum 25(OH)D levels in COVID-19 patients are associated with a more severe disease course [3, 6]. Vitamin D deficiency is one of many factors involved in determining the outcome of COVID-19 disease that can be corrected safely and cheaply [3]. The current study agrees with the discoveries of previous studies [17, 31] found that elevated serum IL-5 levels were linked to poor disease outcomes. To our knowledge, few short-term case studies (i.e., less than two months) in winter have measured the association between serum 25(OH)D levels and acute phase COVID-19 disease severity following in-patient admission [26, 32, 33]. There were also not studies that have clarified the mechanism by which 25(OH)D status affects disease severity of COVID-19 [32].

This study further analyzed the impact of serum 25(OH)D levels on the clinical characteristics of COVID-19 patients. Patients were divided into three groups based on their serum 25(OH)D level: a normal group, a vitamin D insufficient group, and a vitamin D deficient group. Only 29 patients (7.3%) had normal 25(OH)D status, while 15.3% and 77.4% were insufficient and deficient, respectively. These low levels of 25(OH)D might be because this study was conducted in winter when the incidence of acute upper respiratory virus infection is high. Other studied groups that traditionally exhibit vitamin D deficiency or insufficiency, such as older adults, tend to stay at home because of cold weather and the pandemic. These are also the populations that are most vulnerable to COVID-19. This study found that 20% and 16% of vitamin D deficient and vitamin D insufficient patients, respectively developed critical disease. This may be the result of 25(OH)D levels that were unable to provide enough substrate for effective intracrine conversion to the active form of vitamin D, 1, 25(OH)_2_D_3_ [21]. The levels of D-dimer and IL-5 were higher in patients with vitamin D deficiency than in those in the vitamin D normal and insufficient groups, while the number of CD8^+^ T cells was significantly lower in the vitamin D deficient group. Except for COVID-19 patients with diabetes, this study found no significant difference in the levels of coronary heart disease and hypertension by 25(OH)D status. Singh, et al. [34] showed that there is a shared pathophysiologic relationship between diabetes and COVID-19 infection which is more obvious in the presence of vitamin D deficiency. The potential mechanisms need to be further explored.

The logistic analysis results reported here are in agreement with those of Karonova et al. [6] that low 25(OH)D levels are associated with a severe course of COVID-19 and poor prognosis. These findings suggest that vitamin D insufficiency may be a contributing factor, due to the lack of sunlight during the winter months that limits outdoor activity and the opportunity to receive sufficient levels of vitamin D. Other studies have linked disease severity to older age [12]. The findings of the current study are consistent with those of Han et al. [35] and Sun et al. [36] in that LDH and CD8^+^ T cell counts are independent predictors of disease severity in COVID-19 patients. However, studies of whole blood are needed to more fully understand the mechanism that links vitamin D status to COVID-19 disease severity as well as any corresponding serological markers. Results from the current study show that serum IL-5 expression is an independent risk factor for the severity of COVID-19. IL-5 levels were higher in patients with vitamin D deficiency than in those with normal or insufficient vitamin D. A few studies have suggested that anti-IL-5 drugs can reverse aberrant immune responses, and thus protect infected subjects from severe COVID-19-related complications [31, 37]. However, it remains unknown whether vitamin D can affect COVID-19 severity by regulating IL-5.

Studies have indicated a possible relationship between serum 25(OH)D levels and COVID-19 disease outcomes. Bilezikian et al. [38] found that individuals with 25(OH)D levels ≥ 38 ng/mL had a two-fold lower risk of viral acute respiratory infections than those with levels < 38 ng/ml. Other studies have also observed a link between lower concentrations of 25(OH)D and a higher risk of acute respiratory infections [21, 39]. The current study identified the 25(OH)D level that was able to predict COVID-19 mortality as < 36.04 ng/mL. When combined with IL-5 levels and Eos counts, the predictive value was even higher, indicating the advantage of using a 25(OH)D level < 36.04 ng/mL combined with an IL-5 level > 1.70 pg/mL and an Eos count > 0.015 in place of 25(OH)D alone to predict COVID-19-related death. The current study also found that peripheral blood Eos counts, IL-5 levels, and 25(OH)D levels alone should be considered when predicting the risk of death. A very large, multi-center study conducted by Ling et al. [3] found a reduced risk of mortality in acute COVID-19 in-patients who received cholecalciferol treatment, regardless of baseline serum 25(OH)D levels.

Serum 1,25(OH)_2_D_3_ is also active in signaling cascades that promote innate antiviral immune responses, including induction of the antimicrobial peptide, CAMP/LL37, which was originally characterized for its antibacterial properties. Cytokines are important markers of infection and immune status. Interestingly, COVID-19 patients with severe disease also had a marked Th2 immune response concurrent with a cytokine storm. Increased IL-5, IL-13, and immunoglobulin E (IgE) levels were observed in these patients, which also correlated with the severity of the clinical course [40]. This is consistent with the upregulation of IL-5 observed in the critical and deceased patients in the current study. Our findings further showed that 25(OH)D levels in the serum of patients with COVID-19 correlated negatively with the expression of IL-5. Previous studies [18, 41, 42] found that vitamin D supplementation can reduce the levels of IL-5 in patients with asthma and COPD. Thus, it is possible that vitamin D reverses disease in COVID-19 patients by reducing IL-5 production. Vitamin D supplementation is a promising low-cost, low-risk method of controlling COVID-19. The serum 25(OH)D status correlated positively with the CD8^+^ T cell counts, suggesting that vitamin D is involved in regulating the immune response of COVID-19 patients. The specific mechanism for this association is worth further exploration.

As with all retrospective studies, there were several limitations to this study. For example, data distribution was somewhat heterogeneous since not every patient had information available for all studied biomarkers. Power may have been improved with more data values. In addition, this study only included hospitalized patients with known COVID-19 diagnoses. A longitudinal analysis of outcomes is needed to assess whether vitamin D status is also associated with the risk of developing SARS-CoV-2 infection and to identify any long-term sequelae of deficient vitamin D status during acute disease. Finally, the role of vitamin D supplementation requires further study using randomized controlled studies, both to establish its efficacy and to determine its optimal dose and duration of treatment.

## Conclusions

The current study showed that most COVID-19 patients have vitamin D deficiency, and vitamin D status is associated with the severity of COVID-19 during the acute infection phase. Serum 25(OH)D levels combined with IL-5 levels and Eos counts could serve as predictors of early COVID-19-related lung injury and mortality. The levels of 25(OH)D in the serum of patients with COVID-19 correlated negatively with the expression of IL-5, however, the potential mechanism requires further exploration.

## Data Availability

The datasets used and/or analyzed during the current study are available from the corresponding author on reasonable request.

## Abbreviations

ACE2: Angiotensin-converting enzyme 2
BMI: Body mass index
COVID-19: Coronavirus disease 2019
CT: Computed tomography
CRP: C-reactive protein
Eos: Eosinophil
ICU: Intensive care unit
LDH: Lactate dehydrogenase
OR: Odds ratio
PB: Peripheral blood
RAS: Renin-angiotensin system
SpO_2_: Oxygen saturation
SARS-CoV-2: Severe acute respiratory syndrome coronavirus 2
